# Serum immunoglobulin or albumin binding single-domain antibodies that enable tailored half-life extension of biologics in multiple animal species

**DOI:** 10.3389/fimmu.2024.1346328

**Published:** 2024-01-30

**Authors:** Michiel M. Harmsen, Bart Ackerschott, Hans de Smit

**Affiliations:** ^1^ Wageningen Bioveterinary Research, Wageningen University & Research, Lelystad, Netherlands; ^2^ Research and Development, Smivet B.V., Wijchen, Netherlands

**Keywords:** half-life, pharmacokinetics, single-domain antibody, albumin, immunoglobulin, FcRn, VH/VL interface

## Abstract

Single-domain antibody fragments (sdAbs) can be isolated from heavy-chain-only antibodies that occur in camelids or the heavy chain of conventional antibodies, that also occur in camelids. Therapeutic application of sdAbs is often complicated by their low serum half-life. Fusion to sdAb that bind to long-lived serum proteins albumin or IgG can prolong serum half-life of fusion partners. Such studies mostly focused on human application. For half-life prolongation in multiple animal species novel species cross-reacting sdAb are needed. We here describe the isolation from immunized llamas of sdAbs G6 and G13 that bound IgG of 9-10 species analysed, including horse, dog, cat, and swine, as well as sdAb A12 that bound horse, dog, swine and cat albumin. A12 bound albumin with 13 to 271 nM affinity dependent on the species. G13 affinity was difficult to determine by biolayer interferometry due to low and heterogeneous signals. G13 and G6 compete for the same binding domain on Fab fragments. Furthermore, they both lack the hallmark residues typical of camelid sdAbs derived from heavy-chain antibodies and had sequence characteristics typical of human sdAbs with high solubility and stability. This suggests they are derived from conventional llama antibodies. They most likely bind IgG through pairing with VL domains at the VH-VL interface rather than a paratope involving complementarity determining regions. None of the isolated sdAb interfered with FcRn binding to albumin or IgG, and thus do not prevent endosomal albumin/IgG-sdAb complex recycling. Fusions of albumin-binding sdAb A12 to several tetanus neurotoxin (TeNT) binding sdAbs prolonged the terminal serum half-life in piglets to about 4 days, comparable to authentic swine albumin. However, G13 conferred a much lower half-life of 0.84 days. Similarly, in horse, G13 prolonged half-life to only 1.2 days whereas A12 fused to two TeNT binding domains (T6T16A12) had a half-life of 21 days. The high half-life of T6T16A12, which earlier proved to be a highly potent TeNT antitoxin, further supports its therapeutic value. Furthermore, we have identified several additional sdAbs that enable tailored half-life extension of biologicals in multiple animal species.

## 1 Introduction

Many types of proteins, including antibodies, cytokines and hormones are important for therapeutic treatment and/or prevention of various diseases. Antibody therapy increasingly relies on small antibody fragments such as camelid single domain antibodies (sdAbs or VHHs) or single-chain variable fragments (scFv). Due to their molecular weight below 50 kDa they are cleared from the body within several minutes to hours after administration. This poor pharmacokinetics (PK) profile hampers their efficacy. The PK follows two phases, a rapid initial decline due to extravasation into the tissues and a slower terminal elimination phase where antibodies are removed from the body by catabolism and renal filtration. Several methods have been developed to increase the terminal half-life of antibody fragments. This includes chemical modification by PEGylation (1, 2), genetic fusion to albumin (3–5) or Fc domains (6), and fusion to antibodies (7–11), peptides (2) or bacterial proteins (12) binding to long-lived serum proteins such as albumin or immunoglobulin (13). These latter methods have the additional advantage of indirectly recruiting binding to the neonatal Fragment crystallizable (Fc) receptor (FcRn) that is responsible for recycling of albumin and IgG after endocytosis by cells of the reticuloendothelial system, which occurs mainly in the liver. FcRn binds to the Fc portion of IgGs and albumin at acidic pH below 6.5 but not at neutral pH (10, 14, 15). This enables FcRn to rescue these ligands from lysosomal degradation, as binding readily occurs in acidified endosomes and ceases when exposed to the physiological pH of the extracellular surroundings at the cell surface. FcRn deletion in mice results in an about 4-fold decrease in IgG and albumin half-life (14–16).

Early investigations of human therapy with murine monoclonal antibodies (mAbs) revealed a serum half-life of only 1–2 days (17). This much shorter half-life as compared to human IgG (23 days) results from the low affinity of human FcRn for murine IgG. It was later shown that human FcRn shows high affinity for human, rabbit, and guinea pig IgG, but little or no affinity for mouse, rat, bovine, or sheep IgG (13). Human serum albumin (HSA) fusion proteins were similarly found to bind well to human and monkey FcRn but not to mice and rat FcRn (18). Using fusions to IgG or albumin binding sdAbs for serum half-life extension has the advantage of broader species applicability. Thus, sdAbs cross-reacting to human and mouse albumin are applicable for human therapy while allowing studies in mice also (9, 11). A further advantage of using sdAbs for targeting is their small size enabling a more efficient recombinant fusion protein production and better tissue penetration as compared to the more complex Fc or albumin.

In addition to conventional antibodies composed of 2 heavy and 2 light chains, camelids also produce heavy-chain-only antibodies with monomeric variable domains that we refer to as VHH or sdAb. Similar to conventional VHs, VHHs have three highly variable complementarity determining regions (CDRs) that form the paratope involved in antigen binding (19). The second framework region (FR2) of VHHs most often has aliphatic or hydrophilic residues at positions that contain highly conserved hydrophobic residues in conventional VHs involved in binding VL domains. Conventional VHs typically have amino acid residues V42, G49, L50 and W52 [VGLW tetrad; IMGT numbering (20), corresponding to Kabat positions 37, 44, 45 and 47] while sdAbs have F/Y42, E/Q49, R/C50 and F/L/G52 (21–23). VHHs can be classified in different subfamilies based on presence of an additional disulphide bond and different sequence patterns in frameworks. VHH subfamily 1 lacks additional disulphide bonds and contains F42, E49, R50 and F52 (22). Remarkably, when isolating antigen binding VHHs by phage display, occasionally VH domains are isolated that contain the VGLW tetrad. These either originate from genuine heavy-chain-only antibodies lacking a CH1 domain involved in light chain binding, that we refer to as conventional-like VHHs while others originate from conventional antibodies containing a CH1 domain (24), that we refer to as (conventional) VH domains (25) or sdAbs. We use VHHs for sdAbs that (most likely) originate from heavy-chain-only antibodies. Conventional-like VHH domains often have mutation W118R (22, 26) that will undoubtedly disrupt VH-VL interaction due to increasing hydrophilicity of the VH-VL interface (27). Most VHHs, genuine and conventional-like, are highly homologous to the human VH3 gene family. However, occasionally conventional-like VHHs homologous to the VH4 gene family are isolated (28). Isolated conventional VH domains binding antigen in the absence of VL often tend to denature and aggregate (29), which can be reduced by ‘camelization’ through mutations G49E, L50R, and W52G or W52I in the VGLW tetrad (30, 31). However, such camelization is not always successful (31, 32). Several later studies have shown that introduction of acidic amino acids, especially Asp, but also Glu, at different positions in CDR1 reduces aggregation and increases expression and solubility of human VH domains expressed without VL (33–36). The folding back of CDR3 over the former VL interface is also earlier implicated in reducing hydrophobicity of isolated VH (35, 37) and VHH (19, 38, 39) domains.

Most antibody therapeutics are developed for human application. The high costs of antibody therapeutics are often prohibitive for veterinary applications. The companion animals horse, dogs and cats are considered more suited for antibody therapies. A monoclonal antibody for the control of pain in dogs and cats (40) is now authorized for market introduction. We earlier isolated VHH-type sdAbs against tetanus neurotoxin (TeNT) for application in horse. A genetic fusion of the TeNT binding sdAbs T6 and T16 proved a highly potent TeNT antitoxin (41). We here describe the isolation and characterization of camelid sdAbs against IgG and albumin for use in serum half-life extension of fusion partners in primarily dogs and horse, but also cats. We analysed the serum half-life in piglets of several TeNT binding sdAbs fused to albumin binding sdAb A12 or G13 specific for IgG fragment antigen binding (Fab), in comparison with a sdAb against porcine IgG light chain, VI-4, that was earlier shown suitable for serum half-life extension (8). Furthermore, we analysed serum half-life in horse of two multimers containing either A12 or G13 sdAbs. The albumin binding sdAb showed serum half-life prolongation comparable to albumin/IgG half-life but the IgG binding sdAb showed much shorter terminal half-lives of about 1 day. We hypothesize that the IgG binding G13 originates from a conventional llama antibody heavy chain and binds to Fab by mispairing to VL domains through its VH-VL interface region. A12 is a promising tool for serum half-life extension of biologics in horse, dogs, cats and swine while G13 enables tailored PK of biologics in possibly all species producing IgG.

## 2 Materials and methods

### 2.1 Animals

All experiments using animals were done according to EU Directive 2010/63/EU and the Dutch Law on Animal Experiments. For immunization, two (animals 9236 and 9237) 2-year-old female llamas (*Lama glama*) were kept in a meadow and provided with food and water ad libitum. Permission for llama immunization was granted by the Dutch Central Authority for Scientific Procedures on Animals (Permit Number: AVD40100201545). The specific study protocol for immunization of the two llamas of this study (2015005c) was approved by the Animal Welfare Body of Wageningen Bioveterinary Research.

The serum half-life of sdAbs was measured after intramuscular administration in two experiments, using either 24 piglets of about 10 kg (6 weeks old) or 6 Shetland horses of 100-145 kg and 4-7.5 years old. Piglets and horses were kept in stables and provided with food and water ad libitum. Permission for measurement of serum-half-life of sdAbs in animals was granted by the Dutch Central Authority for Scientific Procedures on Animals (Permit Number: AVD401002016655). The specific study protocols for measurement of sdAb serum half-life in piglets (2016.D-0037.001) or horse (2016.D-0037.003) were approved by the Animal Welfare Body of Wageningen Bioveterinary Research.

### 2.2 Antigens and antibodies

The antigens and antibodies used are described in **Supplementary Table 1**. The IgGs from Jackson Immunoresearch Laboratories were obtained as Chrompure IgG or Gamma Globulin IgG. The more highly purified Chrompure IgG was used in llama immunization. During phage display, cat albumin was immobilized to ELISA plates by coating with goat anti-cat albumin IgG and capture of albumin from normal cat serum. In later experiments for analysing binding by yeast-produced sdAbs, purified cat albumin (Equitech-Bio, Inc., Kerrville, TX) was used.

### 2.3 Phage display selections

The immunization of llamas 9236 and 9237 with a commercial tetanus vaccine and a recombinant C-terminal tetanus toxin fragment C on 0, 21 and 42 days post primary immunization (DPPI) and subsequent phage display library generation from blood samples taken at 28 and 49 DPPI was earlier described (41). Simultaneous with these antigens these llamas were also immunized with albumin and (Chrompure) IgG from dog and horse (**Supplementary Table 1**), using 500 µg of each of these 4 antigens per llama per immunization. Phage display selections were performed by biopanning with either albumin from dog and/or horse, or IgG from dog and/or horse. Albumin or IgG were presented by passive adsorption to polystyrene 96-well plates in 50 mM NaHCO_3_ buffer (pH 9.6) at 1 µg/ml. Bound phages were eluted by 30 min incubation at 37°C with 1 mg/ml trypsin in phosphate-buffered saline (PBS) and transduced to *Escherichia coli* TG1 [(F′ traD36 proAB lacIqZ ΔM15) supE thi-1 Δ(lac-proAB) Δ(mcrB-hsdSM)5(rK−mK−)] cells. In each selection round, a phage enzyme-linked immunosorbent assay (ELISA) was performed simultaneously with the phage display selection for evaluation of the phage display. After panning individual colonies were picked and the sdAb genes were induced with 1 mM isopropyl β-d-thiogalactopyranoside.

### 2.4 ELISAs

Several ELISA procedures were used. We first describe the general approach. Polystyrene 96-well ELISA plates (Greiner, Solingen, Germany) were coated with antigens or antibodies/sdAbs in coating buffer (50 mM sodium carbonate buffer, pH 9.6) or PBS, using 100 µl/well, overnight at 4°C. Subsequent incubations were done at room temperature for about 1 hour, followed by washing plates before each next step and using either ELISA buffer (0.5 M NaCl; 2.7 mM KCl; 2.8 mM KH_2_PO_4_; 8.1 mM Na_2_HPO_4_; pH 7.4); containing 1% milk and 0.05% Tween-20 (EBTM) or PBS containing 1% milk and 0.05% Tween-20 (PBSTM). After the last incubation with a horse radish peroxidase (HRP) conjugate the bound HRP was visualized by colour reaction with 3,3`,5,5`-tetramethylbenzidine (Thermo Fisher Scientific, Rockford, IL). The reaction was stopped with sulfuric acid the absorbance at 450 nm (A450) was measured using a Spectramax 340 (Molecular devices) spectrophotometer.

#### 2.4.1 Analysis of *E. coli*-produced sdAbs

Individual *E. coli*-produced sdAb clones were screened in an ELISA with coated albumins, IgGs or IgG fragments at 5 µg/ml in coating buffer. Plates were incubated with sdAbs present in ten-fold diluted *E. coli* culture supernatants. Bound sdAb was detected using 0.5 µg/ml anti-myc clone 9E10 mAb HRP conjugate (Roche Applied Science, Penzberg, Germany).

#### 2.4.2 Analysis of yeast-produced sdAbs

Binding of yeast-produced sdAbs to different albumins or IgGs was analysed using plates coated with 5 µg/ml albumins, IgGs or IgG fragments in coating buffer. Plates were then incubated with 2-fold dilution series of sdAbs starting at 1 µg/ml sdAb concentration in EBTM. Bound sdAb was detected using 0.5 µg/ml 9E10 mAb HRP conjugate.

The ability of sdAbs to bind independent antigenic sites was studied by blocking/competition ELISA using biotinylated sdAbs. ELISAs were performed using plates coated with 5 µg/ml albumins or IgGs in coating buffer. The optimal concentration of biotinylated sdAb for competition was first determined by titration of biotinylated sdAb without competition. For competition, a biotinylated sdAb concentration that provided an absorbance value of 1 was used while unlabeled sdAbs were used at a concentration of 5 µg/ml. Coated plates were first incubated with the unlabeled sdAb in 90 µl/well for 30 min (blocking step). Then 10 µl biotinylated sdAb was added and incubated for another 30 min (competition step). A control without antigen and a control without competing (unlabeled) sdAb were included. Bound biotinylated sdAb was detected by incubation with 0.5 µg/ml streptavidin-HRP conjugate (Jackson Immunoresearch Laboratories). The % inhibition of antigen binding due to a competing sdAb was calculated as 100–100* ([A450 with competing sdAb - [A450 without antigen coating])/([A450 without competing sdAb] - [A450 without antigen coating]).

#### 2.4.3 Analysis of tetanus titres in horse sera

ELISA plates were coated with 0.5 µg/ml TeNT in PBS and then subsequently incubated with EBTM (blocking step), three-fold serial dilution series of horse sera with five-fold starting dilution in EBTM, and anti-horse IgG-HRP conjugate (Jackson Immunoresearch Laboratories). Normal horse serum and a horse anti-tetanus serum were included as negative and positive controls, respectively.

#### 2.4.4 SdAb quantification in piglet or horse sera

TeNT binding sdAb multimers were analysed using plates coated with 2 µg/ml TeNT in PBS whereas M8ggsVI4q6e was analysed using plates coated with 5 µg/ml M23F in coating buffer. The latter plates were subsequently incubated with 5 µg/ml FMDV strain O1 Manisa antigen in EBTM. The TeNT- or M23F/FMDV-coated plates were then incubated with 2-fold dilution series of piglet or horse sera starting at 5-fold dilution over 8 wells. Two dilution series of the sdAb used for injection starting at 1 and 0.1 µg/ml sdAb were included to generate a standard curve. The sdAbs bound to TeNT-coated plates were detected using an anti-his6 monoclonal antibody clone BMG-his1-HRP conjugate (Roche Applied Science) whereas the M8ggsVI4q6e sdAb bound to M23F/FMDV coated plates was detected using 0.5 µg/ml anti-myc clone 9E10 mAb HRP conjugate (Roche Applied Science). A four parameter logistic curve was fitted to absorbance and antibody concentrations of the standards using the SOFTmax Pro 2.2.1 program (Molecular Devices). The serum sdAb concentration was then determined by interpolating the A450 values in the standard curve.

### 2.5 Sequence analysis and sdAb modelling

The sdAb encoding regions were sequenced and aligned according to the IMGT numbering system of the mature sdAb encoding region, ending at sequence VTVSS (20). SdAbs were classified into different CDR3 groups based on having different CDR3 length or less than 65% sequence identity in CDR3. Modelling of sdAb 3D structures was done using deep-learning models for predicting structures of antibodies (42) by accessing the Nanobodybuilder 2 website. 3D structures were rendered using PyMOL 2.5.2 software (Schrodinger, Portland, USA).

The sequences of alpaca (A0A6I9IHM8), bovine (P02769), cat (P49064), dog (P49822), horse (P35747), human (P02768), mouse (P07724), sheep (P14639) and swine (P08835) albumin from the UniProt database were reverse translated to DNA sequences using EMBOSS Backtranseq - Translate Protein sequence to DNA with Mammalian High order (https://www.ebi.ac.uk/Tools/st/emboss_backtranseq/) and aligned using ClustalW (43). Model selections were performed using the function ‘modelTest’ available with the library ‘phangorn’ in R (44) based on the lowest Akaike Information Criterion (45) and Bayesian Information Criterion to identify the best substitution models (Markov models) for DNA sequence evolution that describe changes over evolutionary time. Of the 92 models considered for selection, the Generalised Time Reversible (GTR) model (46), with gamma distributed sites (G4), was found suitable for comparing the different albumin sequences. A GTR phylogenetic tree with gamma-distributed sites was drawn using the library ‘phangorn’ in R (44).

### 2.6 Production of sdAbs

The production of monomeric TeNT binding sdAbs T2L, T6L, T15L and T16L in baker’s yeast strain W303-1a (ATCC number 208352) with C-terminal c-myc and his6 tags was earlier described (41). They were purified from culture supernatant using immobilized-metal affinity chromatography (IMAC) and biotinylated as described recently (47). SdAbs expressed in this manner with C-terminal myc and his6 tags are indicated by the suffix “L”. Novel isolated sdAbs binding IgG (G#) or albumin (A#) were produced as monomeric sdAbs in a similar manner. Monomeric M23F binding FMDV (48) was produced similarly but contained only a C-terminal his6 tag.

We earlier described the production and purification of multimeric sdAbs T2A12, T6A12, T15A12, T16A12, T6T16A12, T2G13, T6G13, T15G13 and T16G13 containing only a C-terminal his6 tag (41) and M8ggsVI4q6e (49) that contains both c-myc and his6 tags. SdAb multimers were produced in yeast strain SU50 using MIRY integrants and purified by IMAC as well as subsequent cation exchange chromatography using SP Sepharose columns.

### 2.7 Biolayer interferometry measurements

The Octet RED96 System (Sartorius, Fremont, CA) was used for affinity measurement based on biolayer interferometry (BLI). An assay temperature of 30°C and a kinetics buffer of PBS containing 0.05% Tween-20 (PBST) were used. The albumin binding affinity was determined using Ni-NTA sensors (Sartorius) that were loaded with sdAbs for 900 sec, and PBST for 300 sec (baseline step). Then association of serial dilutions of albumins from cat, horse, dog or swine (analyte) was done for 60 to 180 sec and finally dissociation for 60 to 300 sec. The Fc binding affinity was determined by loading Ni-NTA (horse Fc) or HIS1K (dog Fc) sensors (Sartorius) with sdAbs for 900 sec, and PBST for 300 sec (baseline step). Then association of serial dilutions of Fc from horse (Fitzgerald Industries) or dog (Rockland Immunochemicals) was done for 60 to 180 sec and finally dissociation for 300 to 600 sec. The Fab binding affinity was determined by loading SAX sensors (Sartorius) with biotinylated sdAbs for 600 sec, and PBST for 300 sec (baseline step). Then association of serial dilutions of dog Fab (Rockland Immunochemicals), starting at 3.3 µM, was done for 200 sec and finally dissociation for 900 sec. The concentrations of analytes and times for association and dissociation were optimized for affinity measurements prior to the experiments. A reference sensor without analyte was included to correct for baseline drift.

The on-rate (*k_a_*) and off-rate (*k_d_*) were determined by global fitting of the association and dissociation phases of a series of albumin concentrations (50). The mathematical model used assumes a 1:1 stoichiometry, fitting only one analyte molecule in solution binding to one binding site on the surface. The equilibrium dissociation constant (*K_D_*), a measure for affinity, was then calculated as the ratio of *k_d_* and *k_a_*. The Octet Analysis Studio v12.2 software (Sartorius) was used for data analysis.

Competition of sdAbs with dog FcRn for binding to sensors loaded with albumin or IgG was also determined by BLI at 30°C. In all steps, a running buffer of 50 mM sodium phosphate pH 5.5 containing 0.05% Tween-20 and an incubation time of 300 sec was used. Biotinylated dog albumin (1 µg/ml), dog IgG (2 µg/ml) or horse IgG (2 µg/ml) was loaded on SAX sensors, followed by a baseline step. The sensors were then incubated with either a sdAb (5 µg/ml; 333 nM) or dog FcRn (10 µg/ml; 200 nM; Thermo Fisher Scientific) to block all sites, followed by a second incubation with the same analyte mixed with another sdAb (5 µg/ml) or dog FcRn (10 µg/ml) as competitor, and an incubation in running buffer again.

### 2.8 SdAb serum half-life determination

Serum half-life of sdAbs was measured in pigs or horse by regular blood sampling during 3-4 weeks. The body weight was determined the day before sdAb injection to allow compensation of sdAb dosing by body weight. SdAbs were filtered through a 0.45 µm membrane before injection in the thigh muscle. Blood samples (5 ml) for serum preparation were allowed to clot for 2 h at 37°C and centrifuged 15 min at 3,000 rpm. Serum samples were stored at -20°C prior to ELISA analysis (Section 2.4.4).

#### 2.8.1 Serum half-life in pigs

Twenty-four piglets were allocated to 4 groups of 6 piglets each that received different sdAbs. Each group consisted of 3 males and 3 females. On day 0 the sdAbs were injected intramuscularly at either 0.2 mg/kg (T6A12), 0.3 mg/kg (T6T16A12) or 0.5 mg/kg (M8ggsVI4q6e, T16A12, T16G13). The M8ggsVI4q6e sdAb was injected into the right thigh of three piglets that also received T6A12 in the left thigh. Blood samples for serum preparation were collected immediately prior to sdAb injection and 1, 2, 4, 8, 11, 14, 21 and 28 days post injection (DPI). The piglet body weight was again determined at 28 DPI to enable compensation of sdAb half-life for body weight gain.

#### 2.8.2 Serum half-life in horse

Six female Shetland horses that were not vaccinated against tetanus were checked for absence of pregnancy by echoscopy and allocated to 2 groups of 3 animals each. The animals were screened for absence of antibody titres against TeNT (Section 2.4.3) prior to inclusion in the experiment. The two groups were intramuscularly injected with either T15G13 or T6T16A12 sdAb at a target dose of 0.17 mg/kg. Blood samples for serum preparation were collected immediately prior to sdAb injection and 1, 2, 4, 7, 10, 13, 17 and 21 DPI.

#### 2.8.3 Terminal serum half-life calculation

PK calculations were performed for individual animals by non-compartmental analysis of serum sdAb concentration versus time data after extravascular injection using PKSolver 2.0 software (51). Since T16G13 and T15G13 serum levels decreased relatively rapidly their half-life was calculated from data obtained from 1 to 4 days. For other sdAb multimers the half-life was calculated from data obtained from 2 to 21 days. The ELISA data obtained from pig serum samples of 28 days DPI were neglected for half-life calculation since the ELISA signals were close to background and as a result possibly relatively high. The half-life of sdAbs in pigs was calculated either directly from measured serum sdAb concentrations or after compensation for body weight gain. The latter was done by multiplying the serum sdAb concentration with the fraction body weight increase that was calculated based on the body weights measured 1 day before and 28 days after sdAb injection and assuming a logarithmic body weight increase over time. Mean values and standard deviation of the values from different animals per group are presented. The concentration observed in time was analysed by a mixed linear regression model, using the nlme library (52) in R (44). The logarithm of the serum sdAb concentration was used as result variable and animal as random variable. Species (pig or horse), time, sdAb multimer and half-life extension sdAb (A12, G13 or VI-4) were used as possible explanatory variables. The best model was selected based on the lowest Akaike Information Criterion (45) using forward selection. Differences of the sdAbs injected in pigs was analysed using the pig data only, to avoid extrapolation.

## 3 Results

### 3.1 Selection of IgG and albumin binding sdAbs

Phage display selection of IgG and albumin binding sdAbs from llama immune libraries was done using IgG and albumin from dog and horse that was directly coated by passive adsorption to polystyrene ELISA plates. For both IgG and albumin 2 rounds of phage display selection were done using dog or horse protein in all 4 possible combinations during the two consecutive rounds. By using different species origin of IgG and albumin in panning round 1 and round 2 we aimed to select for sdAbs that cross-react between the proteins from these species. Simultaneous selections on proteins from the same species during both selection rounds enabled selection of species specific sdAbs as well. Individual clones obtained after two panning rounds were analysed for binding to the proteins derived from horse and dog as well as proteins from several other species to determine their suitability for use with multiple species. Furthermore, sdAb clones selected for binding to IgG were also analysed for binding to Fc or Fab since Fab binding sdAbs are less likely to interfere with effector functions encoded by the Fc fragment such as binding to the neonatal Fc receptor necessary for optimal serum half-life.

After screening and sequencing many clones, 8 unique sdAbs binding IgG (G-clones; **Table 1**) and 6 unique sdAbs binding albumin (A-clones; **Table 2**) were selected for further work. Clones were primarily selected based on their binding to both dog and horse proteins. The IgG binding sdAbs were in addition preferentially selected based on their specificity for Fab fragments of dog or horse. The 14 sdAbs originate from different B cell lineages based on their CDR3 (**Supplementary Figure 1**). The 6 albumin binding sdAbs are all VHHs that belong to VHH subfamily 1 (**Supplementary Figure 1**). VH4 and VH3 gene family sdAbs differ at several positions in FR1, FR2 and FR3. The sdAb G7 showed a high sequence homology to sdAb-31 and sdAb-32 that belong to the VH4 gene family (28). G7 showed different residues to other sdAbs isolated, but identical residues to sdAb-31 and sdAb-32 at framework positions 9, 14, 15, 16, 17, 18, 20, 24, 25, 39, 42, 54, 69, 71, 77, 82, 83, 86, 94 and 95 (**Supplementary Figure 1**). G7 thus belongs to the VH4 gene family. G7 as well as 4 further IgG binding sdAbs contain the ‘VGLW’ motif that is typical of conventional-like VHHs, although they lack the arginine residue at position 118 that is often also present in such VHHs of VH3 family (22, 25, 26) but not of VH4 family (28). Among these conventional-like sdAbs, G6 and G13 contain a leucine residue at position 123 that is associated with reduced production level in yeast (53). G13 contains two Asp residues in CDR1 and G6 contains one Asp residue in CDR1 that could be involved in increasing stability and solubility, similar to isolated human VH domains (34–36). Strikingly, these sdAbs also have substitutions Q120E (G6) or Q120K (G13), which are rarely observed in sdAbs. Since residue 120 is a known interdomain site of VH/VL interfaces (54, 55) these substitutions into more hydrophilic residues could contribute to VH solubilization. The folding back of a long CDR3 over the former VL interface (35, 37–39) could also increase solubility of G6, which contains a long CDR3 of 19 residues with many charged residue, but is less likely the case for G13, which has an 11-residue CDR3. The 3D-structures of G6 and G13 were generated by modelling (42), using VHH-type sdAb A12 as a control. These models confirm the folding back of the G6 CDR3 over the former VL interface (**Supplementary Figure 2**). The 19 amino acids long CDR3 of the A12 VHH similarly folds back over this former VL interface.

**Table 1:** Phage display selection history and binding in ELISA of yeast-produced IgG binding sdAbs.

SdAb	IgG species origin in phage display				Specificity for^a^											SdAb yeast Production level (mg/L)
					Fab/Fc Specificity^b^	Species^c,d^										
	Round 1	Round 2	Llama	DPPI		Ho	Do	Ca	Hu	Bo	Sh	Sw	GP	Mo	Ch	
G3L	Horse	Horse	9237	28	Fc	+	–	–	–	–	–	–	–	–	–	0.29
G6L	Horse	Dog	9236	28	Fab	+	+	+	+	+	+	+	+	+	–	1.8
G7L	Horse	Dog	9237	49	Fab	–	±	–	–	–	–	–	–	–	–	0.15
G13L	Horse	Horse	9236	28	Fab	+	+	+	+	+	±	+	+	+	±	1.6
G18L	Dog	Horse	9237	49	Fab	–	+	±	–	–	–	–	–	–	+	0.30
G19L	Horse	Dog	9237	49	Fab	+	+	+	±	±	–	–	±	±	+	1.6
G23L	Dog	Dog	9237	28	Fc	–	+	–	–	–	–	–	–	–	–	0.41
G24L	Horse	Dog	9237	28	Fc	+^e^	+	–	–	–	–	–	–	–	–	0.99

a Based on ELISA of **Supplementary Figure 3**.

b A450 value above 0.2 on IgG fragment of at least one species is considered indicative of binding.

c +, maximal A450 value >0.4; ±, maximal A450 value <0.4 but above background value; -, A450 values comparable to background values (see **Supplementary Figure 3**).

d Ho, horse; Do, dog; Ca, cat; Hu, human; Bo, bovine; Sh, sheep; Sw, swine; GP, guinea pig; Mo, mouse; Ch, chicken.

e G24L binds to horse IgG since it binds horse Fc with an A450 value above 1 (**Supplementary Figure 3**).

**Table 2:** Phage display selection history and binding in ELISA of yeast-produced albumin-binding sdAbs.

SdAb	Albumin species origin in phage display				Species specificity^a^									SdAb yeast Production level (mg/L)
	round 1	round 2	Llama	DPPI	Ho	Do	Ca	Hu	Bo	Sh	Sw	Mo	Ch^b^	
A2L	Dog	Dog	9237	28	+	+	–	–	–	–	–	±	–	0.22
A4L	Dog	Horse	9237	28	±	–	–	–	–	–	–	±	–	0.18
A6L	Horse	Dog	9237	28	+	+	+	–	–	–	±	±	–	0.21
A7L	Horse	Horse	9237	28	+	–	–	–	–	–	–	–	–	2.82
A12L	Dog	Horse	9237	49	+	+	+	–	–	–	+	–	–	3.85
A16L	Horse	Dog	9237	49	+	+	-^c^	–	–	–	–	–	–	0.73

a Based on ELISA of **Supplementary Figure 4**. See legend of **Table 1** for definitions of specificity and species.

b Chicken ovalbumin.

c A16L binds cat albumin in ELISAs using biotinylated A16L in competition ELISAs or affinity measurements using BLI.

G6 and G13 are both derived from the phage display libraries generated using primer BOLI192 (**Supplementary Figure 1**), which was developed for priming on a heavy-chain antibody isotype identified in llamas with a hinge typified by the GTNEV sequence (8). This oligonucleotide has a relatively high chance of also priming on VH domains since it also has a 12 residue 3´-end priming on the FR4 region of sdAbs, encoding amino acids TVSS, that is conserved in VHs. Taken together, these results suggest that G6 and G13 are VH domains rather than conventional-like VHHs. Furthermore, the three further Fab binding sdAbs are conventional-like VHHs (subfamily C) whereas all Fc or albumin binding sdAb are VHHs having the typical hallmark residues in FR2 (**Supplementary Figure 1**).

### 3.2 Species specificity of yeast-produced sdAbs in ELISA

The 8 IgG binding and 6 albumin binding sdAbs were produced by secretory yeast expression with C-terminal c-myc and his6 tags, as indicated by the suffix “L”. The IgG and albumin binding sdAbs were titrated in ELISA on plates coated with IgG or albumin derived from various species, as well as Fab and Fc fragments of IgG from dog and horse, guinea pig Fc and swine F(ab’)_2_ (**Supplementary Figures 3, 4**). In these experiments chicken ovalbumin should be considered a negative control since it shows less than 15% sequence identity to the albumins used. The maximal absorbance value obtained as well as the extent to which IgG binding sdAbs can be diluted before reaching background absorbance values varies considerably between individual sdAbs and is also dependent on species origin of IgG used. Furthermore, background absorbance values without sdAb were slightly elevated up to 0.269 using sheep IgG, chicken IgG and especially using horse IgG (**Supplementary Figure 3**). Therefore, a maximal absorbance value of >0.4 was considered indicative of binding to an IgG for each species (+ sign in **Table 1**). However, an absorbance value just below 0.4 that is nevertheless above background absorbance values is observed for some sdAb/IgG combinations (± sign in **Table 1**), that are all Fab specific. G3L only binds horse IgG.

The binding to horse, dog or swine IgG fragments consistently showed that G3L, G23L and G24L bound Fc whereas the further 5 sdAbs bound Fab (**Table 1**). Binding to IgG fragments is mostly consistent with binding to whole IgG from a particular species. A notable exception is G24L that clearly binds horse Fc but did not bind horse IgG (**Supplementary Figure 3**).

All six sdAbs bound to horse albumin in ELISA, although the ELISA signals obtained varied considerably (**Supplementary Figure 4**). A12L and A16L showed the highest ELISA signals, with absorbance values above 1.5 and still showing binding at VHH concentrations below 0.01 µg/ml. These sdAbs also bound well to dog albumin. A12L also bound swine and cat albumin. A6L also bound albumins from the important target species horse and dog and in addition bound swine, mouse and cat albumin (**Supplementary Figure 4**). The binding to albumins from different species is summarized in **Table 2**. The cross reaction to albumins of different species is consistent with the albumin amino acid sequence homology (**Supplementary Figure 5**), that shows close homology of dog and cat albumin as well as bovine and sheep albumin while mouse albumin shows closest homology to horse albumin.

SdAb G13 was selected for further work because it is specific for Fab and bound IgG of all species (**Table 1**). G6 also bound Fab, and bound to all mammalian IgGs, but not chicken IgG (**Table 1**). G7 was ignored for further work because of its low ELISA signal and low yield from yeast expression (**Table 1**). A6, A12 and A16 were similarly selected because of their high ELISA signals on horse and dog albumin and broad species reactivity (**Supplementary Figure 4**). Furthermore, clones G13 and A12 were both relatively well produced in yeast (**Tables 1**, **2**).

**Figure 1:**
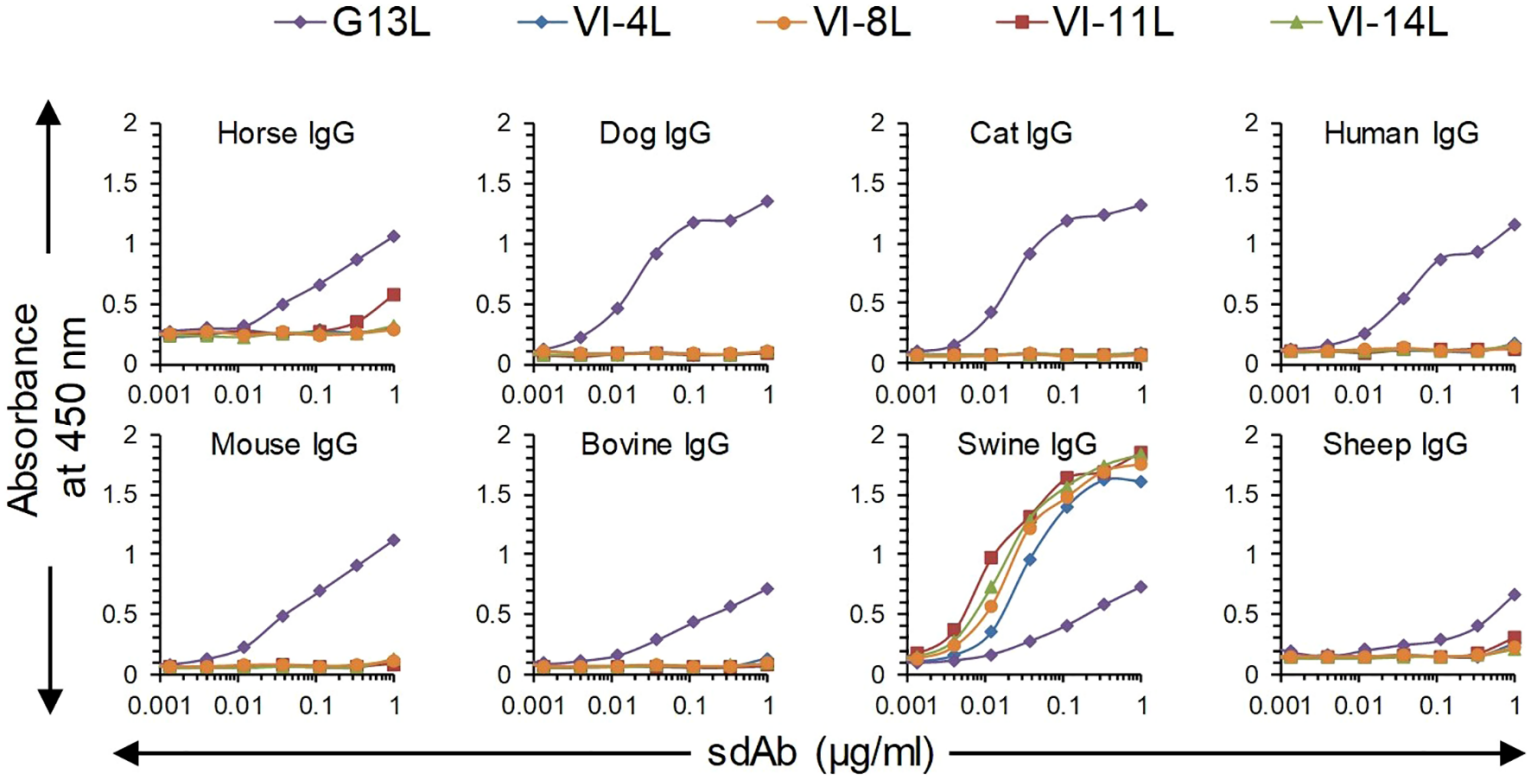
Comparison of broadly reactive sdAb G13L with four earlier isolated sdAbs specific for swine IgG in ELISA. Plates coated with IgG from eight different species were incubated with threefold dilution series of sdAbs, which were subsequently detected using an anti-myc HRP conjugate. G13L shows a less sigmoidal curve as compared to the other four sdAbs, including three sdAbs against F(ab’)_2_ (VI-4L, VI-8L and VI-14L).

We earlier isolated sdAbs VI-4L, VI-8L and VI-14L, which bind swine IgG and F(ab’)_2_ fragments, and VI-11L, which only binds swine IgG. These sdAbs bind highly efficiently to swine IgG without cross-reaction to IgGs from 7 further mammalian species in case of VI-4L, VI-8L and VI-14L, and only weakly cross-react to horse IgG in case of VI-11L (**Figure 1**). Furthermore, these four sdAbs had a more sigmoidal ELISA curve and higher absorbance values as compared to G13L, which showed increasing absorbance values until the highest sdAb concentration tested using swine, horse, mouse and bovine IgG (**Figure 1**).

### 3.3 Epitope binning

**Figure 2:**
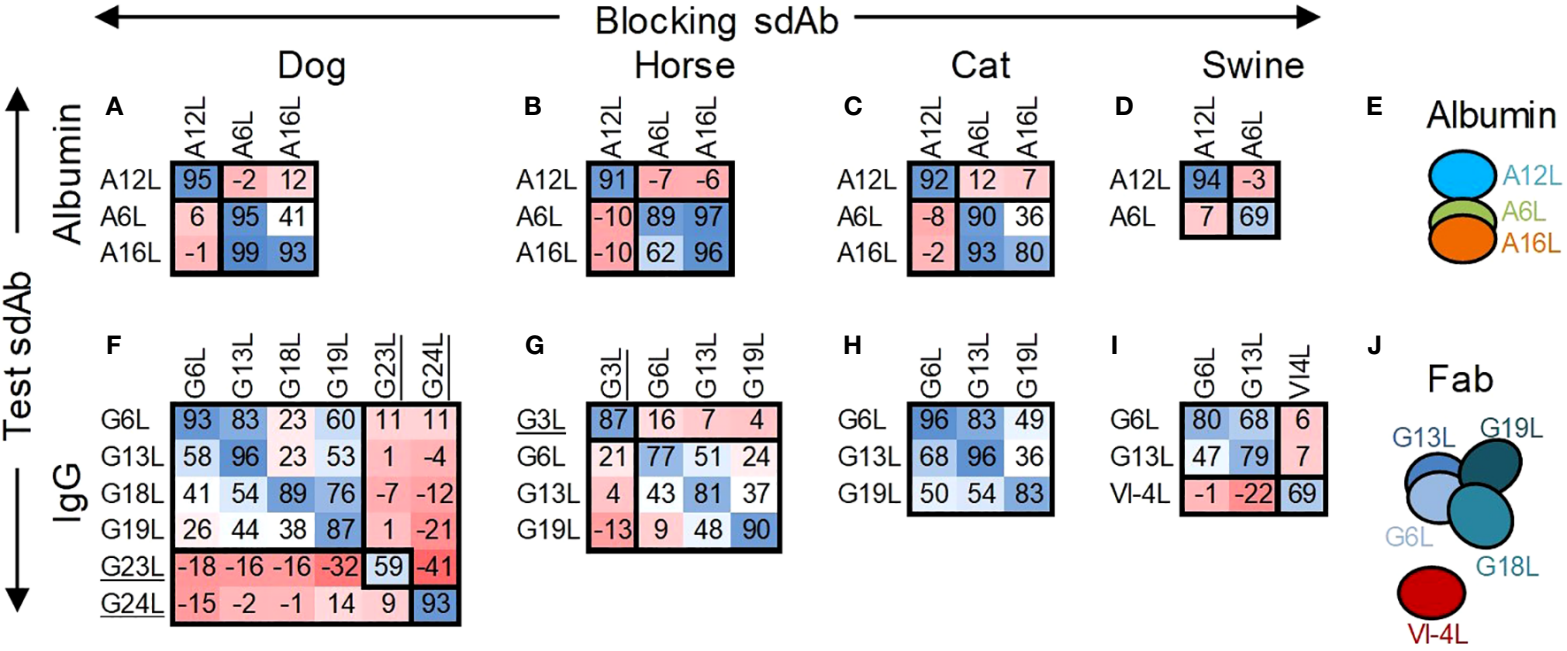
Epitope binning of albumin and IgG binding sdAbs by competition ELISA. The percentage inhibition by unlabelled blocking sdAbs of binding of a biotinylated test sdAb to directly coated albumins **(A-D)** or IgGs **(F-I)** of dog, horse, cat or swine is indicated using a blue/red colouring scheme. The deduced allocation of sdAbs to antigenic sites on albumins **(E)** or Fab fragments **(J)** is indicated by ovals that overlap to varying degrees. The Fc binding sdAb are underlined.

Epitope binning of the three selected albumin binding sdAbs and all IgG binding sdAbs except G7 was done by competition ELISAs using horse, dog, cat and swine antigens (**Figure 2**). Here, we used biotinylated test sdAbs that were competed with unlabelled sdAbs. Contrary to the ELISAs done using an anti-myc mAb HRP conjugate (**Table 2**), the biotinylated A16L was also found to react with cat albumin, although with relatively low absorbance value. A12L and A6L clearly recognized a separate antigenic site on all four albumins while A16L competed reciprocally with A6L using horse, dog and cat albumin (**Figures 2A-D**). The four Fab-binding sdAb, G6L, G13L, G18L and G19L, competed with each other for binding to dog IgG (**Figure 2F**), although the percentage inhibition varied. They showed >87% inhibition when the same sdAb was used for blocking and competition, but lower inhibition when using heterologous sdAb combinations. This pattern was also observed using G6L, G13L and G19L on horse IgG and G6L and G13L on cat and swine IgG (**Figures 2G-I**). G6L and G13L competed more efficiently with each other than with G18L or G19L (**Figures 2F-H**). The Fab binding sdAb VI-4L, which is specific for swine IgG, did not compete with G6L or G13L (**Figure 2I**). As expected, the Fc binding sdAbs G3L, G23L and G24L did not compete with any of the Fab binding sdAb (**Figures 2F, G**). G23L and G24L recognized independent sites on dog IgG. The interpretation of antigenic sites recognized by sdAb against albumin and Fab is schematically represented in **Figures 2E, J**.

### 3.4 SdAb competition with FcRn

**Figure 3:**
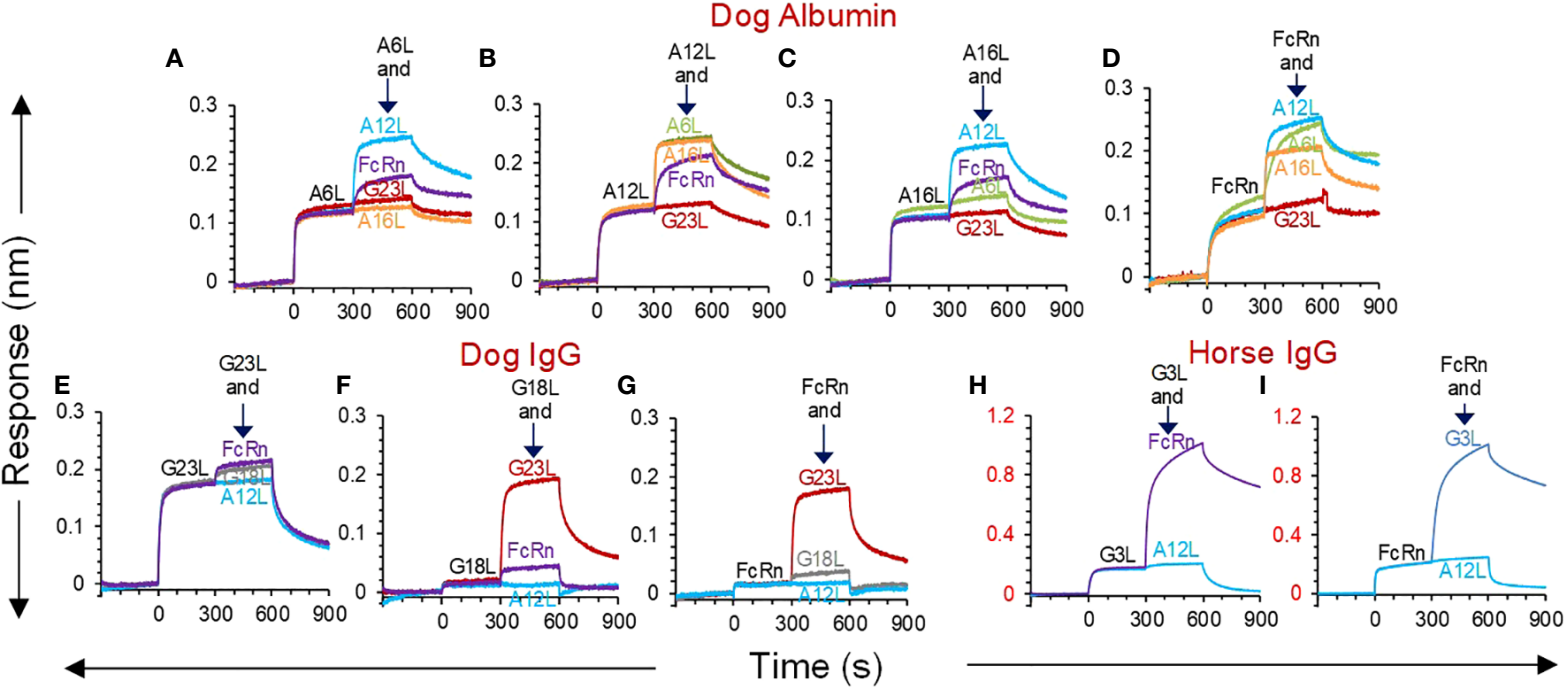
BLI analysis of competition between sdAbs and dog FcRn for binding to albumin or IgG. SAX biosensors were loaded with biotinylated dog albumin (**A-D**), dog IgG **(E-G)** or horse IgG **(H, I)** ligands, followed by a baseline step. At time = 0 sec, sdAbs or dog FcRn analytes were allowed to associate with the coupled ligands in a running buffer at pH 5.5, which allows FcRn binding. At time = 300 sec, a second association was done using the same analyte as in the first association (black text), that was mixed with a second analyte (coloured text). Each panel represents different sensorgrams of the same first analyte mixed with different second analytes. The different panels using the same ligand represent reciprocal competitions. None of the sdAbs competes with FcRn while competition between sdAbs is consistent with epitope binning by ELISA. G23L and A12L were used as negative control VHHs for binding to albumin and IgG, respectively.

Competition between FcRn and sdAbs for binding to albumin or IgG was analysed using BLI. A commercially available dog FcRn was used to measure interaction at pH 5.5 with biotinylated dog albumin, dog IgG or horse IgG as ligands loaded on sensors. The sdAb and FcRn concentrations used resulted in saturation binding to these ligands during a first incubation of 300 s. This was followed by a second incubation with the same analyte, mixed with a second analyte. A response during this second association step indicates independent binding of the two mixed analytes. A6L, A12L and A16L did not compete with FcRn, while competition between sdAbs only occurred using A6L and A16L (**Figures 3A-D**), confirming earlier epitope binning by ELISA. Since the FcRn binding site on IgG resides on the Fc fragment we only analysed competition with Fc binding sdAb G3L, which is specific for horse IgG, and G23L, which is specific for dog IgG, and used G18L, which is Fab specific, as a control. Both G3L, G18L and G23L do not compete with FcRn (**Figures 3E-I**). Remarkably, G18L gave a much lower response than G23L (**Figures 3E-G**) or the albumin binding sdAbs (**Figures 3A-D**). Even lower responses were observed with the other three broadly reactive Fab binders (results not shown).

### 3.5 SdAb affinities

The affinity of the three selected sdAbs for horse, dog, cat and swine albumins was determined by BLI (**Table 3**). The equilibrium dissociation constant (*K_D_*) varied from 1.2 to 271 nM depending on the nature of the sdAb and albumin. A12L had relative low affinity (high *K_D_*) for dog and swine albumin (>= 189 nM) but higher affinity for horse and cat albumin (<= 31 nM). The affinities of A6L and A16L for different albumins were in the range of 1.2 to 13 nM.

**Table 3:** Affinity of sdAbs for albumin of different species measured by BLI.

SdAb	*K_D_* (nM)				*k_d_* × 10^-5^ (1/s)			
	Horse	Dog	Cat	Swine	Horse	Dog	Cat	Swine
A6L	13	3.2	1.2	ND^a^	966	226	87	ND
A12L	13	271	31	189	115	1250	82	2489
A16L	11	13	2.0	ND	242	510	29	ND
T6T16A12	0.65	268	ND	ND	14	620	ND	ND

a ND, not determined.

The affinity of Fc binding sdAbs G3L, G23L and G24L was determined in a similar manner using sdAbs immobilized to Ni-NTA or HIS1K sensors binding to horse or dog Fc analyte. The curves fitted well to the 1:1 stoichiometry interaction model (R^2^ > 0.98), suggesting that the 0.24-4.5 nM *K_D_* values were reliable (**Table 4**). However, in similar experiments the Fab-specific sdAbs G6L, G13L, G18L and G19L did not show detectable binding of Fabs (results not shown). When using SAX sensors for coupling of biotinylated sdAbs, binding of dog Fab could be detected for G13L, G18L and G19L, but not G6L (**Supplementary Figure 6**). These three sdAbs showed low ELISA responses, similar as observed with G18L in FcRn competition experiments (**Figures 3E-G**). They appeared to have average affinities (*K_D_* = 38 to 70 nM). However, they showed weak correlation with a 1:1 interaction model (R^2^ < 0.94; **Table 4**), indicating that the *K_D_* values measured are unreliable. The poor curve fitting of the Fab binding sdAb contrasted with the good curve fitting observed with Fc or albumin binding sdAb (**Supplementary Figure 6**). This could indicate heterogeneous binding to Fab.

**Table 4:** Affinity of sdAbs for Fab or Fc determined by BLI.

SdAb	Analyte	*K_D_* (nM)	*k_a_* × 10^5^ (1/Ms)	*k_d_* × 10^-5^ (1/s)	R^2 a^
G6L	Dog Fab	Binding not detectable			NA^b^
G13L	Dog Fab	70	0.31	220	0.916
G18L	Dog Fab	38	0.49	187	0.911
G19L	Dog Fab	62	0.32	197	0.941
G3L	Horse Fc	0.68	8.4	58	0.980
G23L	Dog Fc	0.24	23	55	0.992
G24L	Horse Fc	4.5	0.86	39	0.997
G24L	Dog Fc	2.7	1.4	37	0.992

a A correlation coëfficient (R^2^) above 0.95 is considered a good fit of the fitted and experimental data (50).

b NA, not applicable.

### 3.6 A12 and G13 sdAb multimers

**Figure 4:**
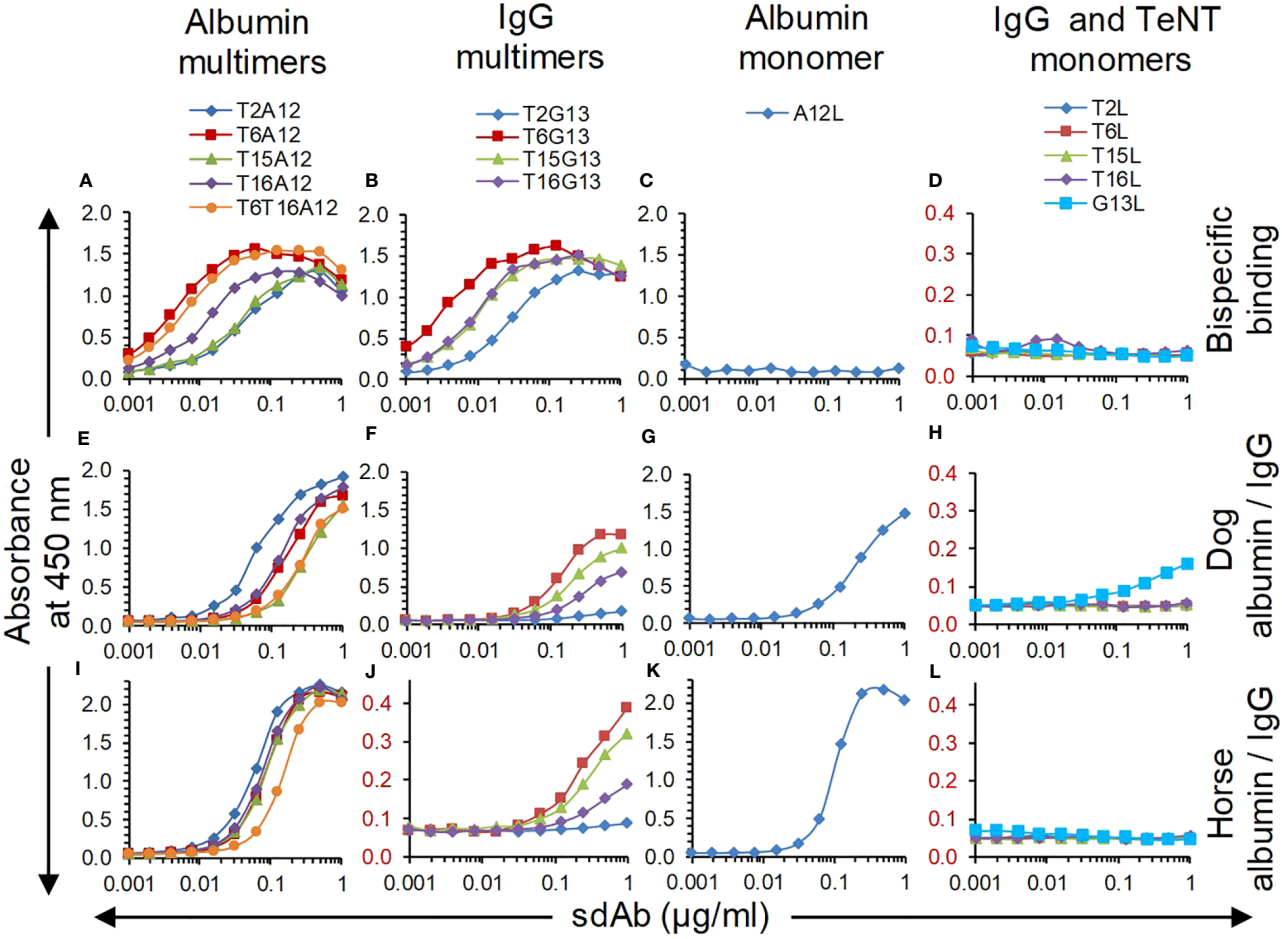
Analysis of sdAb multimer binding in ELISA, using sdAb monomers as control. **(A-D)** Bispecific binding was analysed using plates coated with dog albumin **(A, C)** or dog IgG **(B, D)**, that were subsequently incubated with twofold dilution series of sdAbs, biotinylated TeNT and streptavidin-HRP conjugate. Binding to dog albumin **(E, G)**, horse albumin **(I, K)**, dog IgG **(F, H)** or horse IgG **(J, L)** was determined by direct coating of these antigens and subsequent incubation with twofold dilution series of sdAbs and anti-his6 mAb-HRP conjugate.

Eight multimers of either G13 or A12 with TeNT binding sdAb domains T2, T6, T15 and T16 were earlier produced in yeast using flexible GGGGSGGGS linkers to join the N-terminal TeNT binding sdAbs with the C-terminal G13 or A12 sdAbs. Furthermore, a trimer was produced that contains T6 and T16 fused by a (GGGGS)_3_ linker that was further similarly linked to C-terminal A12 (41). These multimers were analysed for simultaneous binding to TeNT and either albumin or IgG from dog using a sandwich ELISA setup requiring bispecific binding to these antigens. All sdAb multimers bind to both TeNT and either albumin in case of A12 containing multimers (**Figure 4A**) or IgG in case of G13 containing multimers (**Figure 4B**) whereas monomeric sdAbs do not show binding in these ELISAs (**Figures 4C, D**). All five A12 multimers as well as A12L monomer bound to directly coated dog (**Figures 4E, G**) and horse albumin (**Figures 4I, K**). Binding of G13 multimers to dog (**Figure 4F**) or horse IgG (**Figure 4J**; note the extended Y axis) was also observed, although with lower absorbance values as compared to albumin and also clearly lower titres as compared the bispecific ELISA (cf. **Figures 4B, F, J**). Furthermore, binding of monomeric G13L to dog IgG resulted in an A450 value of at most 0.161 which was however above the background absorbance observed with 4 sdAbs against TeNT (**Figure 4H**) while binding to horse IgG was not observed (**Figure 4L**). This is at least partly due to the use of an anti-his6 mAb-HRP conjugate for sdAb detection since the ELISA signals obtained using the myc-tag for sdAb detection were generally much higher (**Supplementary Figure 3**). Furthermore, T6T16A12 bound dog and horse albumin with an affinity that was equal or increased as compared to monovalent A12L (**Table 3**). Thus, all multimers have retained capacity to bind to albumin or IgG despite fusion of TeNT binding sdAb domains. A12 containing multimers had high absorbance values and sigmoidal curves whereas G13 containing multimers had relatively lower ELISA signals, similar to results obtained with monovalent sdAbs.

### 3.7 Serum half-life

**Figure 5:**
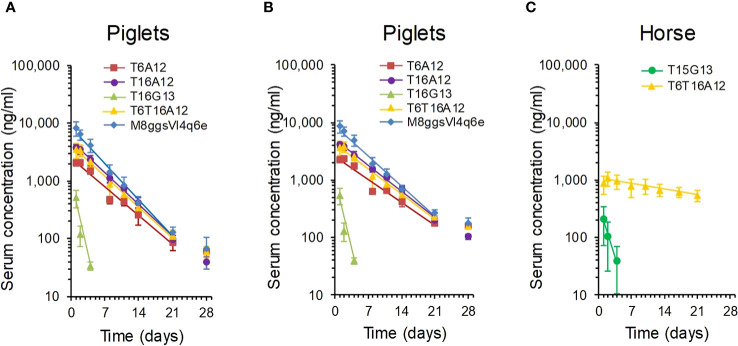
Analysis of serum half-life of multimeric sdAbs binding to albumin or IgG. Multimeric sdAbs containing either VI-4 or G13 domains binding to IgG or containing an A12 domain binding to albumin were intramuscularly injected into groups of piglets **(A, B)** or horses **(C)** and serum sdAb concentration was analysed by ELISA over time. The measured serum sdAb concentrations were either presented directly **(A, C)** or compensated for piglet body weight gain **(B)**. Data points represent average and standard deviation of 3 piglets (M8ggsVI4q6e), 6 piglets (4 TeNT binding multimers) or 3 horses. The straight line was fitted for calculation of terminal serum half-life.

We first measured the terminal serum half-life of intramuscularly bolus injected sdAbs in 6-week-old piglets. We used the T16G13 multimer binding to IgG and 3 multimers containing the albumin binding A12 sdAb, including the T6A12 and T16A12 dimers and the T6T16A12 trimer. The serum sdAb concentration was measured using a TeNT specific ELISA. Three out of 6 piglets that received T6A12 also received the FMDV binding M8ggsVI4q6e that could be separately analysed in serum samples using an FMDV specific ELISA. The serum sdAb concentration of the 3 albumin binding sdAbs as well as M8ggsVI4q6e decreased logarithmically during the 28 days of blood sampling at similar rates (**Figure 5A**). Their terminal serum half-lives were calculated from 2 to 21 DPI since the ELISA values obtained at 28 DPI were generally close to background signals and the sdAb concentration at 1 DPI is part of the extravasation phase (8). They had terminal half-lives of 3.3 to 4.2 days. The T16G13 serum concentration decreased much more rapidly. It could barely be detected in ELISA at 4 DPI and was consistently absent in samples from 8 DPI or later (**Figure 5A**), resulting in a half-life of 0.84 days. Piglets at age of 6 to 10 weeks generally grow fast. The individual piglets had a 2.0 to 3.0-fold increase in body weight during this experiment. Therefore, the measured sdAb concentration was compensated for body weight gain also (**Figure 5B**). The serum half-lives, measured with and without body weight gain compensation, are summarized in **Table 5**.

**Table 5:** Summary of terminal serum half-life of sdAb multimers in piglets and horse.

SdAb	Species	Admini- stration Route^a^	No. of Animals Per Group	SdAb Dosing	Terminal serum half-life (days)^b^		
					Without Weight gain Compensation	With Weight gain Compensation	Reference
K609ggsK812	Piglets	i.v.	4	0.2 mg/kg	0.08 ± 0.004	ND^c^	(8)
K609ggsVI4q6e	Piglets	i.m.	3	0.5 mg/pig	7.1 ± 1.1	ND	(8)
M8ggsVI4q6e	Piglets	i.m.	3	0.5 mg/kg	3.3 ± 0.11	4.0 ± 0.14	This work
T6A12	Piglets	i.m.	6	0.2 mg/kg	4.2 ± 0.36	5.2 ± 0.53	This work
T6T16A12	Piglets	i.m.	6	0.3 mg/kg	3.9 ± 0.23	4.8 ± 0.34	This work
T16A12	Piglets	i.m.	6	0.5 mg/kg	3.8 ± 0.33	4.6 ± 0.48	This work
T16G13	Piglets	i.m.	6	0.5 mg/kg	0.84 ± 0.15	0.9 ± 0.16	This work
T15G13	Horse	i.m.	3	0.17 mg/kg	1.2 ± 0.65	ND	This work
T6T16A12	Horse	i.m.	3	0.17 mg/kg	21 ± 4.5	ND	This work

a I.v., intravenous; i.m., intramuscular.

b Mean ± standard deviation.

c ND, not determined.

Six horse that were not vaccinated against tetanus were selected for this study. They had low antibody titres against TeNT, comparable to titres in normal horse serum and more than 1000-fold lower titres than a hyperimmunized horse serum (**Supplementary Figure 7**). The serum half-life of T6T16A12 and T15G13 was analysed in 3 horses each. Despite the use of a different TeNT binding sdAb (T15) as compared to the half-life determination in piglets (T16) the G13 containing multimer again showed a low half-life of only 1.2 days. However, the albumin binding T6T16A12 showed a serum half-life of 21 days (**Figure 5C**, **Table 5**).

Statistical analysis of the joint data of the piglet and horse study showed that the serum half-life (decrease in log sdAb concentration over time in the analysis) was significantly longer in horses by a factor 3.4. The serum half-life also differed significantly dependent on the three half-life elongation sdAbs used (A12, G13 and VI-4). The best half-life in pigs was observed with the albumin binding sdAbs. The slight decrease in sdAb half-life of the VI-4 containing multimer as compared to the three A12 containing multimers was significant, as well as the further decrease in half-life of the G13 multimers. Multimers containing Fab binding sdAbs G13 and VI-4 also showed a significant different half-life.

## 4 Discussion

Here, we isolated sdAbs against IgG and albumin of horse and dog by phage display selection from immunized llamas, aiming for species cross-reactive sdAbs, for use in serum half-life extension. Six sdAbs against albumin were obtained which all contained the hallmark residues typical of VHHs that originate from heavy-chain antibodies. SdAbs A6L, A12L and A16L were further characterized since they cross-reacted with horse, dog and cat albumin while A12L also bound well to swine albumin. A6L and 16L had affinities (*K_D_*) for horse, dog and cat albumin ranging from 1.2 to 13 nM while A12L had tenfold lower affinities (higher *K_D_*) ranging from 13 to 271 nM. Due to the high serum concentration of albumin and IgG even proteins that bind with low affinity to these serum proteins can reach a serum half-life equalling albumin or IgG. Many studies on serum half-life extension using different binding proteins or peptides have shown that an affinity below 1 µM can provide a useful PK profile (9, 10, 56–58). This suggests that A12 is suitable for serum half-life extension even in dogs, for which it has the lowest affinity. Furthermore, A12 is probably also suitable for half-life elongation in cats since it binds cat albumin with 31 nM affinity. However, PK is also dependent on binding kinetics, with lower *k_d_* giving better PK and binding at the acidic endosomal pH is most relevant for PK (11) while we measured affinity at neutral pH. Thus, half-life extension using A12 in different species should be studied *in vivo* by animal experiments. A6L and A16L were found to recognize an overlapping antigenic site while A12L recognized an independent site. These sites were again independent of the FcRn binding site on dog albumin suggesting they do no prevent endosomal recycling of albumin-sdAb complexes. Others also observed that most sdAbs against albumin do not interfere with FcRn binding (11).

The terminal serum half-life in swine of two tetanus binding sdAbs (T6 and T16) and a genetic fusion of these two domains (T6T16) that were each genetically fused to an A12 domain ranged from 3.8 to 4.2 days. This is considerably lower than the 7.1 day serum half-life that we earlier measured in piglets (8) using the K609 sdAb fused to the IgG binding sdAb VI-4 (**Table 5**). However, the same VI-4 sdAb, when fused to the M8 sdAb, in the current analysis showed a serum half-life of 3.3 days, which is more similar to the half-life of multimers containing the A12 domain (**Table 5**). The fast growth of piglets also affects the serum half-life. We therefore also calculated serum half-lives by compensating for body weight increase, which resulted in slightly increased half-lives (**Table 5**). Authentic albumin has a 8.2 ± 0.7 days serum half-life in 20-kg piglets (59). Most likely experimental variation causes the different half-lives of authentic albumin and A12 containing sdAb multimers as well as different VI-4 containing multimers. The serum half-life of T6T16A12 in horse was found to be 21 days. Unfortunately we could not find publications describing the albumin half-life in horse. However, the half-life of IgG in horses was reported to be 23 days in newborn foals (60) and 14.3 ± 1.7 days in mature healthy horses (61), which is comparable to the 21-days half-life of A12 multimers.

Species cross-reactive sdAbs against albumin have been isolated earlier by several groups, mostly focusing on cross reaction between human and mouse albumin, to enable use of mouse studies with such sdAbs (9, 62). Recently a large collection of 71 sdAbs against human albumin were isolated, 8 (11%) of which cross-reacted to mouse albumin while none cross-reacted to bovine albumin (11). To our knowledge A6L, A12L and A16L are the first example of horse, dog and cat albumin cross-reacting sdAbs. This facilitates therapeutic use of sdAbs for serum half-life elongation of biologics in these species. We have earlier shown that T6T16A12 potently neutralizes tetanus neurotoxin (41). The good serum half-life of T6T16A12 in horse further supports the use of T6T16A12 for therapeutic application in horses and other species.

We also isolated eight sdAbs against horse and dog IgG, three of which bound to Fc. The Fc binders were specific for either horse, dog or cross-reacted with horse and dog Fc. They were similar to the albumin binding sdAbs in many respects. They had high affinities (*K_D_* = 0.24-4.5 nM), showed sigmoidal ELISA curves and did not compete with FcRn for binding to IgG. Their sequences were typical of VHHs. However, the further five sdAbs against Fab had the VGLW motif in FR2 that is typical of conventional VHs and lacked the W118R mutation that is often observed in conventional-like VHHs that originate from heavy-chain antibodies. This contrasts with our earlier isolation of 19 sdAbs against porcine IgG, which comprised only one conventional-like VHH, which contained the W118R mutation (8). The five novel sdAbs most likely originate from conventional antibodies. Two of them, G6L and G13L, also had acidic residues in CDR1 that are often observed with stable conventional VHs and were isolated using an oligonucleotide that is more likely to prime on conventional VH domains. This further suggests they originate from conventional antibodies. Clone G7 was a VH4 family type sdAb that was not further characterized. The further four Fab-binding sdAbs all recognized an at least partially overlapping antigenic site. However, competition varied from 9% to 96%, dependent on the sdAb combination and IgG species origin. Furthermore, all four sdAbs showed non-sigmoidal curves in ELISA, low BLI signals and indications of heterogeneous binding in BLI. These phenomena all indicate that these sdAbs bind Fab by binding VL domains through their VH-VL interface. Since this requires interaction with different VL domains this explains the heterogeneous binding in BLI and epitope binning. Possibly, G13L shows improved Fab binding as compared to G6 due to its relatively short CDR3 that does not fold back over the VL interface.

Numerous studies have shown that VH-VL pairing in conventional antibodies is promiscuous (63–65) although analysis of a large dataset of antibodies showed that VH–VL pairing does not occur at random (66). Formation of antigen binding Fvs by heterologous pairing of mouse and human variable domains has also been used for humanization of rodent antibodies by guided selection (67, 68). Mispairing of antibody light chains is also often observed during single-cell production of bispecific antibodies (69). This domain pairing promiscuity probably also explains the heterologous pairing of llama VH domains with antibodies from multiple species. Although it is more likely that such pairing occurs with VL domains, the formation of VH homodimers cannot be excluded since such VH homodimers, that are able to interact with antigens, have been described (70, 71). The VH-VL interaction shows variable affinity in different Fvs. The interaction is sometimes strong enough to result in stable Fvs capable of antigen binding (72) while other Fvs show weak interactions that cannot be measured by surface plasmon resonance and result in loss of antigen binding (73). Fvs are often stabilized by introduction of disulphide bonds or a peptide linker to produce scFvs (74, 75), although this is sometimes even insufficient for stabilization and may result in aggregation of scFvs (76).

In two earlier studies IgG binding sdAbs were isolated where the authors did not discuss possible binding based on VH-VL interface interaction while several reported observations support such a mechanism. When panning a naïve VHH library against two scFvs a diverse panel of sdAbs was isolated that were surprisingly all VH domains containing the VGLW tetrad (24). A mechanism based on VH-VL domain interaction would explain the low affinity of scFv binders, diversity of CDR3s of isolated sdAbs and frequent isolation of sdAbs that cross-react with both scFvs. Some of these sdAbs were shown to be linked to CH1 in the original cDNA and thus originate from conventional H2L2 antibodies (24). Similar proof that G13 and four further Fab binding sdAbs of this study are linked to CH1 domains would unequivocally demonstrate their VH nature. In a second study, a human VH domain that was well expressed in bacteria without VL was used as a scaffold for generation of a CDR3 randomized synthetic phage display library. After panning on a mouse monoclonal antibody a panel of sdAbs was obtained with again diverse CDR3 sequences. One sdAb was further characterized. It had 20 nM affinity and cross-reacted with mouse, human, rabbit and hamster IgG and various Ig isotypes (77).

G13 was found to prolong serum half-life to only about 1 day in pigs and horse, which is clearly less than using the albumin binding A12. However, serum half-life is extended as compared to the K609ggsK812 multimer that is unable to bind IgG or albumin and has a 0.08 day half-life in pigs (**Table 5**) (8). It was earlier reported that an albumin binding VHH-type sdAb (M75) showed similar moderate half-life extension in rats of only 3.8 to 6.8 h while other albumin binding sdAb prolonged half-life to more than 40 h, similar to rat albumin (10). M75 bound well to rat albumin at pH 7.4 but not at the acidic pH of the endosome. Furthermore, M75 binding induced albumin conformational changes that prevented interaction with FcRn. This suggests that the M75 moderate half-life is due to prevention of renal filtration without FcRn-mediated prevention of catabolism. Due to the different antibody domains recognized by G13 (Fab) and FcRn (Fc) it is unlikely that conformational changes induced by G13 binding prevent FcRn binding to IgG. Generally, pH-sensitive binding relies on histidine residues that are only positively charged at acidic pH (78). However, histidine residues are absent in G13 (**Supplementary Figure 1**) and do not frequently occur in VL domains. They are absent in 34 out of 113 human germline V lambda domains and most likely do not occur in the hydrophobic VH-VL interfaces. Thus, it is unlikely that G13 shows pH-sensitive binding. Most likely other factors determine the modest serum half-life prolongation by G13. Possibly the putative VH-VL domain association as molecular basis of G13 binding to IgG plays a role. However, other factors have also been implicated in the rate of antibody elimination, including its antigen binding specificity, immunogenicity and susceptibility to proteolysis (13, 15).

Taken together we have obtained several sdAbs against albumin or IgG that enable tailored half-life extension of biologics in horse and swine, and most likely also dogs and cats, and have validated the long half-life of the albumin binding sdAb using a promising tetanus antitoxin in horse.

## Data availability statement

The original contributions presented in the study are included in the article/**Supplementary Materials**. The sequences of isolated sdAbs have been deposited in the Genbank database with accession numbers PP062726 to PP062739. Further inquiries can be directed to the corresponding author/s.

## Ethics statement

The animal study was approved by the Animal Welfare Body of Wageningen Bioveterinary Research. The study was conducted in accordance with the local legislation and institutional requirements.

## Author contributions

MH: Conceptualization, Formal analysis, Investigation, Supervision, Visualization, Writing – original draft, Writing – review & editing. BA: Formal analysis, Investigation, Writing – review & editing. HD: Conceptualization, Funding acquisition, Project administration, Supervision, Validation, Writing – review & editing.
