# Supplementary material for: Serum immunoglobulin or albumin binding single-domain antibodies that enable tailored half-life extension of biologics in multiple animal species
Source: Front Immunol. 2024 Jan 30;15:1346328. doi: 10.3389/fimmu.2024.1346328 (PMC10862077; doi:10.3389/fimmu.2024.1346328)
Supplement: Supplementary file 1 [file DataSheet_1.pdf]

Supplementary Material

Serum immunoglobulin or albumin binding single-domain antibodies that enable tailored half-life extension of biologics in multiple animal species

Michiel M. Harmsen, Bart Ackerschott, Hans de Smit

1 Supplementary Figures and Tables

Supplementary TABLE 1 | Antigens and antibodies used.

Sample	Species	Supplier[a]	Cat. No.
mAbs, pAbs, sera and HRP conjugates			
Anti-myc clone 9E10 mAb HRP conjugate	Mouse	Roche Applied Science	11814150001
Anti-his6 BMG-his-1 mAb HRP conjugate	Mouse	Roche Applied Science	11965085001
Rabbit anti-Goat Ig HRP conjugate	Rabbit	Dako	P160
Streptavidin HRP conjugate	NA	Jackson Immunoresearch Laboratories	016-030-084
Goat anti-llama Ig HRP conjugate	Goat	Bethyl Laboratories	A160-100P
Normal cat serum	Cat	Agrisera Antibodies	AS10 1567
Goat anti-cat albumin IgG	Goat	Antibodies Online	ABIN457608
Rabbit anti-horse IgG HRP conjugate	Rabbit	Jackson Immunoresearch Laboratories	308-035-003
Normal horse serum	Horse	Jackson Immunoresearch Laboratories	008-000-001
Horse anti-tetanus serum	Horse	Intervet International	A037A01
IgGs			
Chrompure IgG	Swine	Jackson Immunoresearch Laboratories	014-000-003
Chrompure IgG	Bovine	Jackson Immunoresearch Laboratories	001-000-003
Gamma Globulin	Cat	Jackson Immunoresearch Laboratories	002-000-002
Gamma Globulin	Chicken	Jackson Immunoresearch Laboratories	003-000-002
Chrompure IgG	Dog	Jackson Immunoresearch Laboratories	004-000-003
Chrompure IgG	Guinea Pig	Jackson Immunoresearch Laboratories	006-000-003
Chrompure IgG	Horse	Jackson Immunoresearch Laboratories	008-000-003
Gamma Globulin	Human	Jackson Immunoresearch Laboratories	009-000-002
Gamma Globulin	Mouse	Jackson Immunoresearch Laboratories	015-000-002
Gamma Globulin	Sheep	Jackson Immunoresearch Laboratories	013-000-002
IgG fragments			
$F(ab')_2$	Swine	Jackson Immunoresearch Laboratories	014-000-006
Fab	Horse	Jackson Immunoresearch Laboratories	008-000-007
$F(ab')_2$	Horse	Jackson Immunoresearch Laboratories	008-000-006
Fc	Horse	Fitzgerald Industries	31C-CH0804
Fc	Guinea pig	Jackson Immunoresearch Laboratories	006-000-008
Fab	Dog	Rockland Immunochemicals, Inc.	004-0105
Fc	Dog	Rockland Immunochemicals, Inc.	004-0103

Supplementary TABLE 1 | Antigens and antibodies used (continued).

Sample	Species	Supplier[a]	Cat. No.
Albumins			
Ovalbumin	Chicken	Sigma Aldrich	A5503
Albumin	Horse	Rockland Immunochemicals, Inc.	008-0133-0010
Albumin	Dog	Molecular Innovations	DSA
Albumin	Human	Jackson Immunoresearch Laboratories	009-000-051
Albumin	Mouse	Antibodies Online	ABIN2131799
Albumin	Sheep	Antibodies Online	ABIN2131803
Albumin	Bovine	Sigma Aldrich	A7906
Albumin	Swine	Antibodies Online	ABIN2131241
Albumin	Cat	Equitech-Bio, Inc.	FSA62
Other proteins			
Tetanus holotoxin	C. tetani	List Biologicals	#190B
FcRn	Dog	Thermo Fisher Scientific	17786348

[a] Agrisera Antibodies, Vännäs, Sweden; Fitzgerald Industries, Tompkinsville, KY; Antibodies Online, Beijing, China; Bethyl Laboratories, Montgomery, TX; Dako, Glostrup, Denmark; Equitech-Bio Inc, Kerrville, TX; Intervet International, Boxmeer, the Netherlands; Jackson Immunoresearch Laboratories, West Grove, PA; List Biologicals, Campbell, CA; Molecular Innovations, Novi, MI; Roche Applied Science, Penzberg, Germany; Rockland Immunochemicals, Limerick, PA; Sigma Aldrich, St Louis, MO; Thermo Fisher Scientific, Rockford, IL.

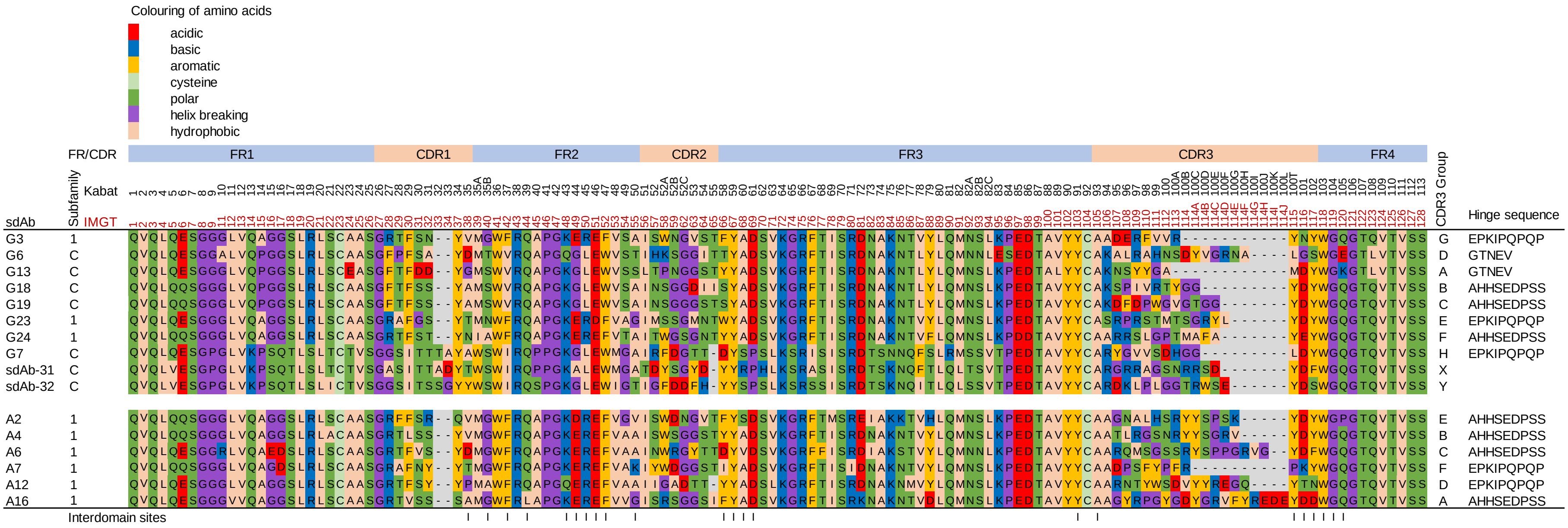

Supplementary Figure 1. Amino acid sequence alignment of yeast-produced IgG or albumin binding sdAbs. SdAb-31 and sdAb-32, that belong to the VH4 gene family are included for comparison with G7. Dashes indicate gaps introduced for sequence alignment. The IMGT system was used for alignment and numbering of sdAbs as well as defining the different complementarity determining regions (CDRs) and framework regions (FR). The Kabat numbering scheme is given for reference. The sdAbs were classified into CDR3 groups (right) and subfamilies (left) as described earlier (1). Amino acids were classified and color-coded based on their physicochemical properties. The interdomain sites of VH/VL interfaces (bottom) were taken from references (2, 3).

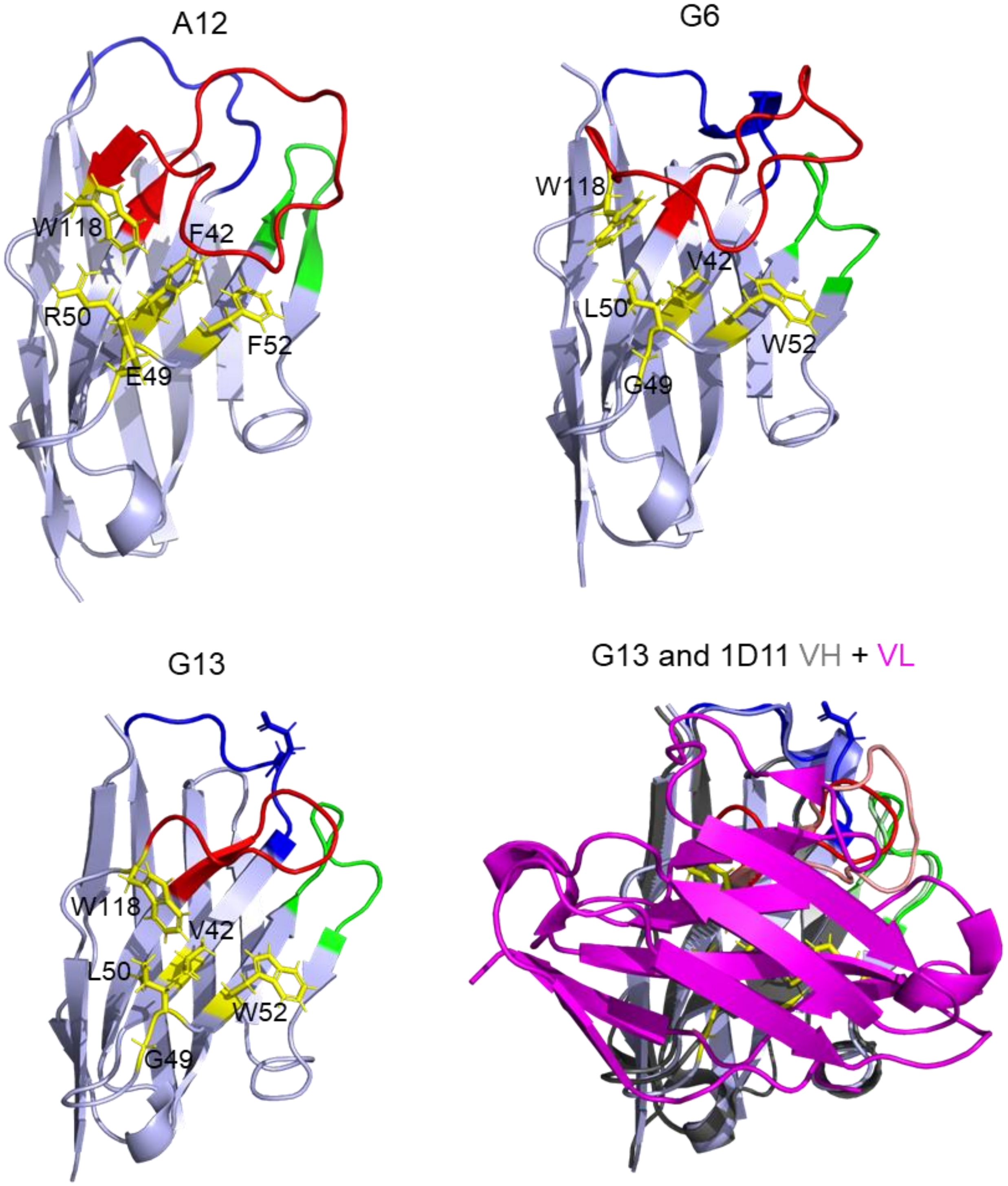

Supplementary Figure 2. Ribbon plots of 3D structures of sdAbs A12, G6 and G13 that were generated by modeling. A search with the G13 sequence in the PDB gave the llama Fab 1D11 (PDB entry 6GLW) as closest homolog with 80% identical VH amino acid sequence. It was aligned with G13 (bottom right) to visualize the position of the VL interface. CDR3, red; CDR2, green; CDR1, blue. CDRs have brighter colors for sdAbs and more pale colors for Fab SD11 VH. VH framework is in light blue (G6, G13, A12) or grey (1D11) while VL is magenta. SdAb residues 42, 49, 50, 52 and 118 (IMGT numbering) are in yellow with side chains shown. Residues 42, 49, 50 and 52 form the VGLW tetrad typical of VH domains as also seen with G6 and G13 while A12 has the FERF tetrad typical of (subfamily 1) VHH domains at these positions.

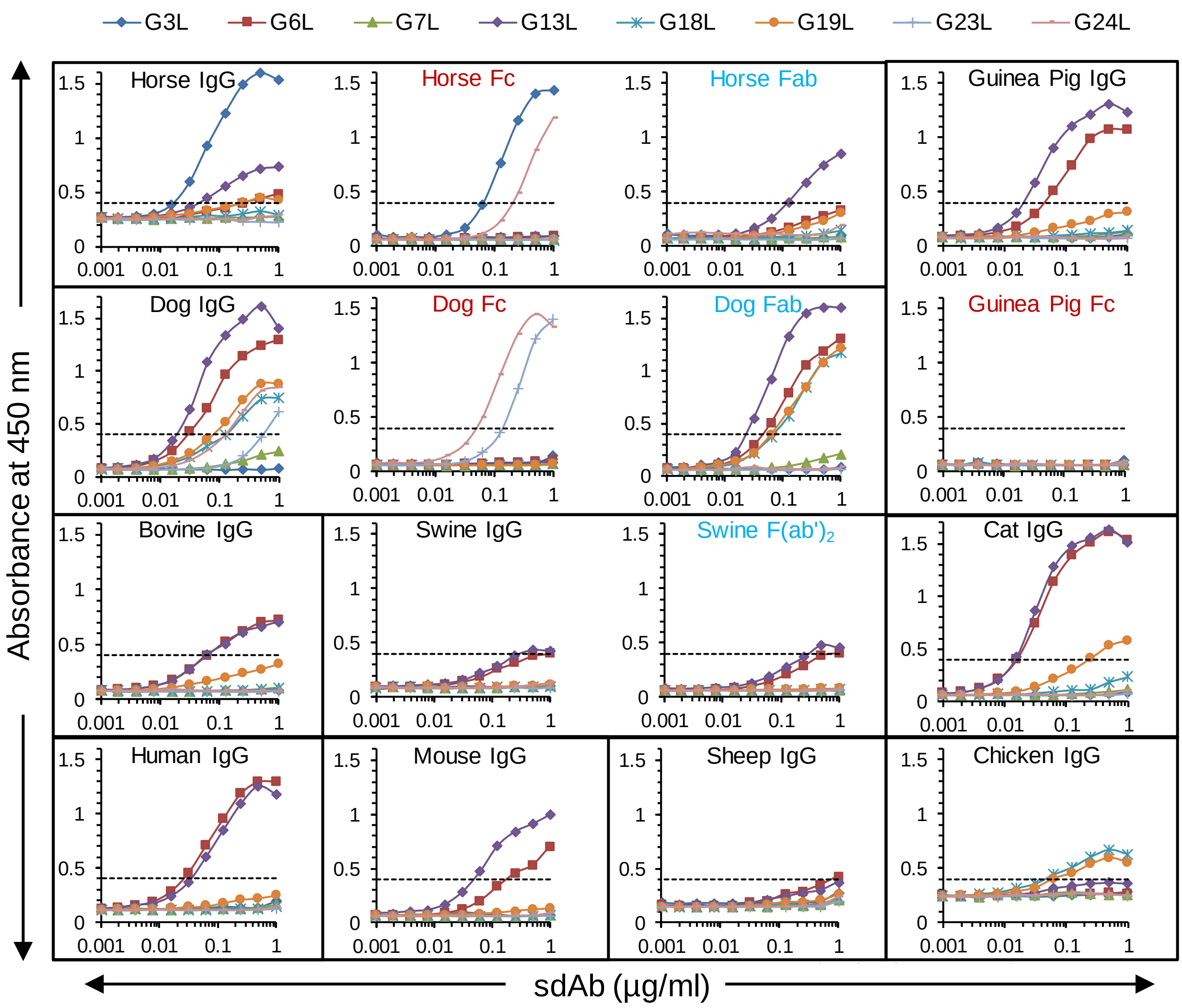

Supplementary Figure 3. Binding of yeast-produced sdAbs to IgGs or fragments thereof of different species in ELISA. Plates coated with IgG (black), Fab or F(ab')$_2$ (blue) or Fc (red) were incubated with twofold dilution series of sdAbs, which were subsequently detected using an anti-myc HRP conjugate. Graphs are boxed according to their IgG species origin. A cutoff A450 value of 0.4 (dashed line) was used to classify species specificity of sdAbs.

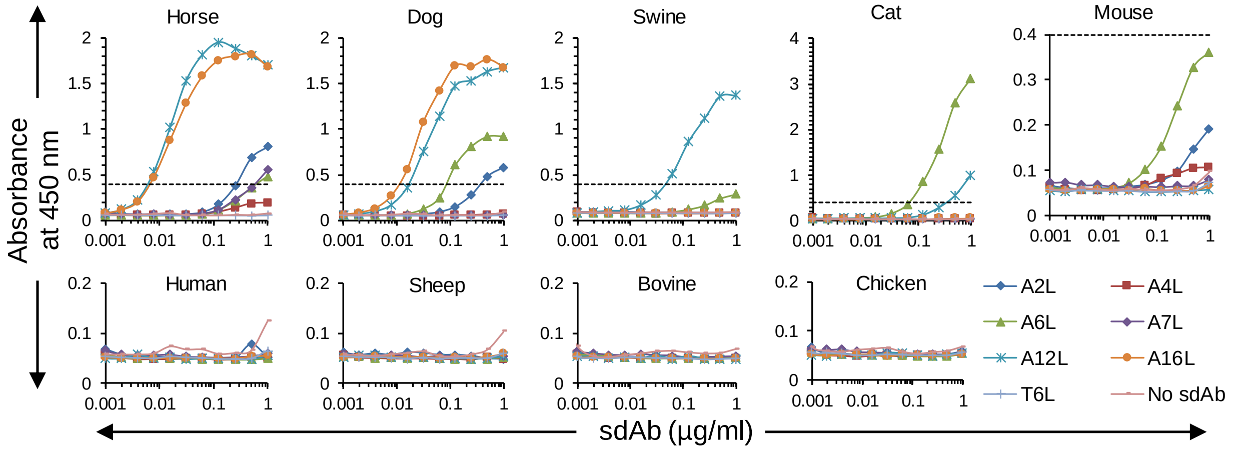

Supplementary Figure 4. Binding in ELISA of yeast-produced sdAbs to different mammalian albumins or chicken ovalbumin. Plates coated with albumins/ovalbumin were incubated with twofold dilution series of sdAbs or without sdAb (No sdAb), which were subsequently detected using an anti-myc mAb HRP conjugate. For some graphs extended Y-axis scales were used to visualize lower ELISA signals. A cutoff A450 value of 0.4 (dashed line) was used to classify species specificity of sdAbs.

A

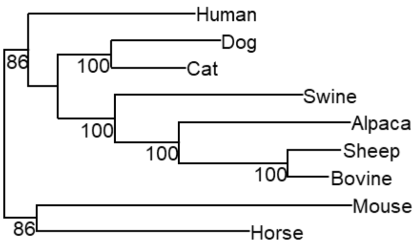

B

Distance	Identity (%)								
	mouse	horse	human	dog	cat	swine	alpaca	sheep	bovine
mouse		71	72	72	73	71	69	69	70
horse	0.29		77	76	78	76	72	76	74
human	0.28	0.23		80	82	76	76	76	77
dog	0.28	0.24	0.20		87	78	77	78	77
cat	0.27	0.22	0.18	0.13		80	77	78	79
swine	0.29	0.24	0.24	0.22	0.20		78	78	80
alpaca	0.31	0.28	0.24	0.23	0.23	0.22		79	80
sheep	0.31	0.24	0.24	0.22	0.22	0.22	0.21		92
bovine	0.30	0.26	0.23	0.23	0.21	0.20	0.20	0.08	

Supplementary Figure 5. Homology of albumin protein sequences. (**A**) Generalised Time Reversible Tree with gamma-distributed sites of protein sequences of the 8 albumins used for evaluating sdAb binding and alpaca albumin as close homolog of albumin from llamas that were used for immunization. The credibility of the tree topology was confirmed by 100 iterations of bootstrap. (**B**) Matrix of percentage identity and distance of albumin protein sequences.

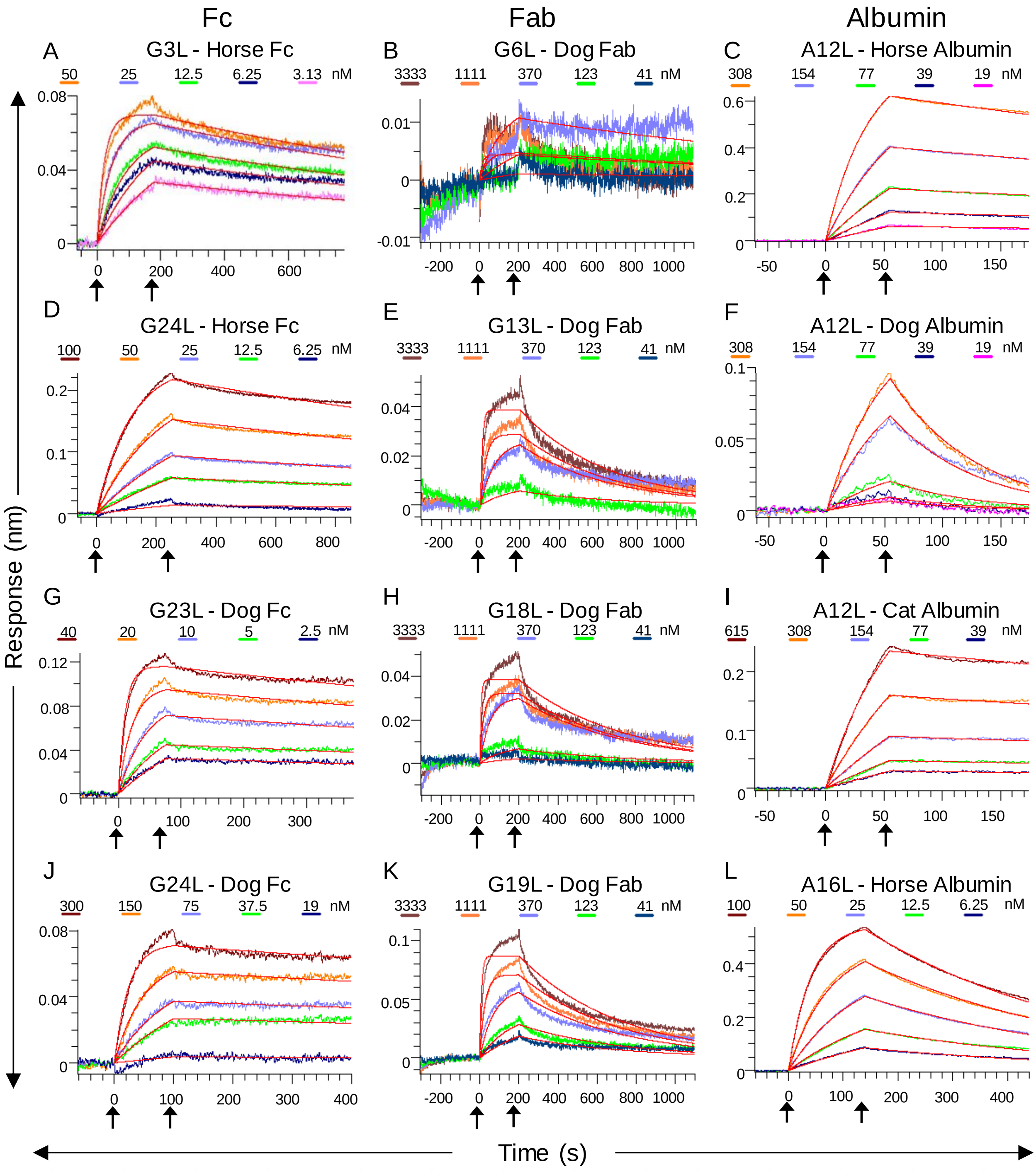

Supplementary Figure 6. Affinity determination of sdAb binding by biolayer interferometry. Individual sdAbs were coupled to Ni-NTA (**A,C,D,F,I,L**), HIS1K (**G,J**) or SAX (**B,E,H,K**) sensors. The sensorgrams show the three incubations of sdAb-loaded sensors, demarcated by arrows, in buffer until t=0 (baseline), association phase with Fc, Fab or albumin (analytes) and dissociation phase in buffer. Each panel shows the interaction of one sdAb with a specific analyte at different concentrations (see legends) that were used for global curve fitting to a 1:1 interaction model (red lines). Fab binding to G6L (**B**) could not be detected while Fab binding to G13L, G18L or G19L resulted in low responses and poor curve fitting (**E,H,K**).

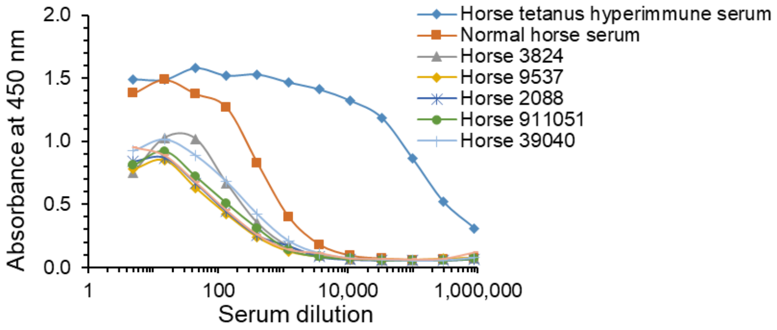

Supplementary Figure 7. Analysis of tetanus titres in horse sera by ELISA. Plates coated with tetanus neurotoxin were incubated with threefold dilution series of horse sera. Bound horse IgG was detected with an anti-horse IgG antibody conjugated to HRP. The horses indicated by a number were used in the serum half-life study whereas the normal horse serum and tetanus hyperimmune serum are negative and positive control sera, respectively.

References

1. Harmsen MM, Ruuls RC, Nijman IJ, Niewold TA, Frenken LGJ, de Geus B. Llama heavy-chain V regions consist of at least four distinct subfamilies revealing novel sequence features. *Mol Immunol.* (2000) 37:579-90. doi: 10.1016/S0161-5890(00)00081-X.

2. Chothia C, Gelfand I, Kister A. Structural determinants in the sequences of immunoglobulin variable domain. *J Mol Biol.* (1998) 278:457-79. doi: 10.1006/jmbi.1998.1653.

3. Chothia C, Novotny J, Bruccoleri R, Karplus M. Domain association in immunoglobulin molecules. The packing of variable domains. *J Mol Biol.* (1985) 186:651-63. doi: 10.1016/0022-2836(85)90137-8.
